# Adverse in-hospital outcomes after shoulder arthroplasty by diabetes status: a retrospective study using the national inpatient sample

**DOI:** 10.3389/fmed.2026.1885571

**Published:** 2026-09-09

**Authors:** Kenan Sun, Liu Yang, Le Fan, Shicheng Liu, Ying Xu, Zifeng Wang, Abudousaimaiti Maimaituxun, Jun Li

**Affiliations:** 1 Department of Orthopedics, The Second Affiliated Hospital of Anhui Medical University, Hefei, China; 2 Institute of Orthopedics, Research Center for Translational Medicine, The Second Affiliated Hospital of Anhui Medical University, Hefei, China; 3 School of Osteopathy, Henan University of Chinese Medicine, Zhengzhou, Henan Province, China; 4 Division of Orthopaedic Surgery, Department of Orthopaedics, Nanfang Hospital, Southern Medical University, Guangzhou, Guangdong, China; 5 Department of Orthopedic Joint, The First Peoples’ Hospital of Kashi Prefecture, Kashi, Xinjiang, China

**Keywords:** nationwide inpatient sample, postoperative complications, total shoulder arthroplasty, type i diabetes mellitus, type II diabetes mellitus

## Abstract

**Background:**

Shoulder arthroplasty is a well-established surgical intervention for osteoarthritis, rotator cuff arthropathy, and complex shoulder diseases. While effective in pain relief and functional improvement, perioperative complications remain a major concern. Patients with diabetes present metabolic disturbances and suboptimal glycemic control, conferring higher risks of adverse perioperative events. Nevertheless, evidence regarding the impact of diabetes subtypes on short-term perioperative outcomes after shoulder arthroplasty remains scarce. This study employed the U.S. National Inpatient Sample (NIS) database to explore the association between diabetes subtypes and perioperative outcomes following shoulder arthroplasty.

**Method:**

Data were derived from the National Inpatient Sample (NIS) database from 2016 to 2022. Patients undergoing shoulder arthroplasty were included and classified into three groups: patients with type 1 diabetes mellitus (T1DM), patients with type 2 diabetes mellitus (T2DM), and patients without diabetes. Patient demographics, hospital characteristics, surgical parameters, comorbidities, perioperative complications, and postoperative outcomes were assessed. Propensity score matching was applied to balance baseline characteristics between groups. Regression analyses were conducted to evaluate postoperative outcomes among patients with different DM subtypes.

**Result:**

A total of 137,651 shoulder arthroplasty cases were identified, comprising 107,388 patients without diabetes, 376 patients with type 1 DM, and 29,887 patients with type 2 DM. Following propensity score matching, multivariate analysis demonstrated no significant between-group differences in most medical and surgical complications when comparing patients without diabetes and patients with type 1 DM. In contrast, type 2 DM was identified as an independent risk factor for pneumonia/acute respiratory failure (OR = 1.19, *P* < 0.001), surgical site/periprosthetic joint infection (OR = 1.25), urinary tract infection (OR = 1.25), acute renal failure (OR = 1.53), non-routine discharge (OR = 1.34), prolonged length of stay (GMR = 1.04), and increased total hospital charges (GMR = 1.02).

**Conclusion:**

Compared with patients without diabetes, patients with type 2 DM undergoing shoulder arthroplasty exhibited higher risks of several adverse in-hospital outcomes, whereas most outcomes in patients with type 1 DM were not significantly different. These findings may facilitate improved risk stratification and tailored perioperative management.

## Introduction

Diabetes mellitus (DM) remains one of the most common chronic metabolic diseases worldwide and continues to impose a substantial public health burden. According to the 11th edition of the International Diabetes Federation (IDF) Diabetes Atlas, approximately 11.11% of adults aged 20 to 79 years, corresponding to about 589 million people, were living with diabetes in 2024 (1). In the United States, type 2 diabetes accounts for the vast majority of diagnosed diabetes cases, whereas type 1 diabetes represents a much smaller proportion. As the prevalence of diabetes continues to rise, the number of patients with DM who may require shoulder arthroplasty is also expected to increase in the coming years (2, 3).

Shoulder arthroplasty is now widely used in the treatment of osteoarthritis, rotator cuff arthropathy, and other complex shoulder disorders, while the number of patients with concomitant DM is also increasing (4). Previous studies of hip and knee arthroplasty have shown that diabetes and its treatment status are associated with an increased risk of postoperative complications, suggesting that patients with DM may face a higher risk of adverse perioperative outcomes (5). Owing to underlying metabolic abnormalities and poor glycemic control, patients with DM may be more susceptible to perioperative complications after shoulder arthroplasty (6, 7). Perioperative complications such as pneumonia, respiratory failure, urinary tract infection, and acute renal failure are important indicators of surgical outcomes and provide valuable information for optimizing perioperative management (8). In addition, existing studies have demonstrated that different DM categories have a significant impact on perioperative outcomes after lumbar fusion surgery (9). However, over the past decade, only limited research has explored whether this distinction is also applicable to patient undergoing procedures other than lumbar fusion (10, 11). The specific effects of different diabetes types on outcomes after shoulder arthroplasty remain unclear.

Using the National Inpatient Sample (NIS) database, this study analyzed the demographic characteristics, hospitalization costs, length of stay (LOS), and comorbidity profiles of patients with type 1 and type 2 DM undergoing shoulder arthroplasty. In addition, we compared patients undergoing shoulder arthroplasty with type 1 or type 2 DM complications with those without complications, with a particular focus on differences in the incidence of perioperative complications among the three groups. This study aims to provide evidence to support surgical management and individualized preoperative risk assessment in patients with type 1 and type 2 DM.

## Methods

### Data source

This study was a retrospective analysis using data from the National Inpatient Sample (NIS) database, a comprehensive inpatient repository managed by the Healthcare Cost and Utilization Project (HCUP) under the Agency for Healthcare Research and Quality (AHRQ). The NIS captures approximately 20% of all inpatient discharges from more than 1,000 hospitals across the United States, yielding a highly representative national sample (12–14).

Patients who underwent shoulder arthroplasty between 2016 and 2022 were identified using relevant International Classification of Diseases, 10th Revision (ICD-10) procedure and diagnosis codes: 0RRJ00Z, 0RRJ07Z, 0RRJ0J6, 0RRJ0J7, 0RRJ0JZ, 0RRJ0KZ, 0RRK00Z, 0RRK07Z, 0RRK0J6, 0RRK0J7, 0RRK0JZ, 0RRK0KZ. The dataset included demographic characteristics, comorbid conditions, and perioperative complications.

### Study population

**Figure 1:**
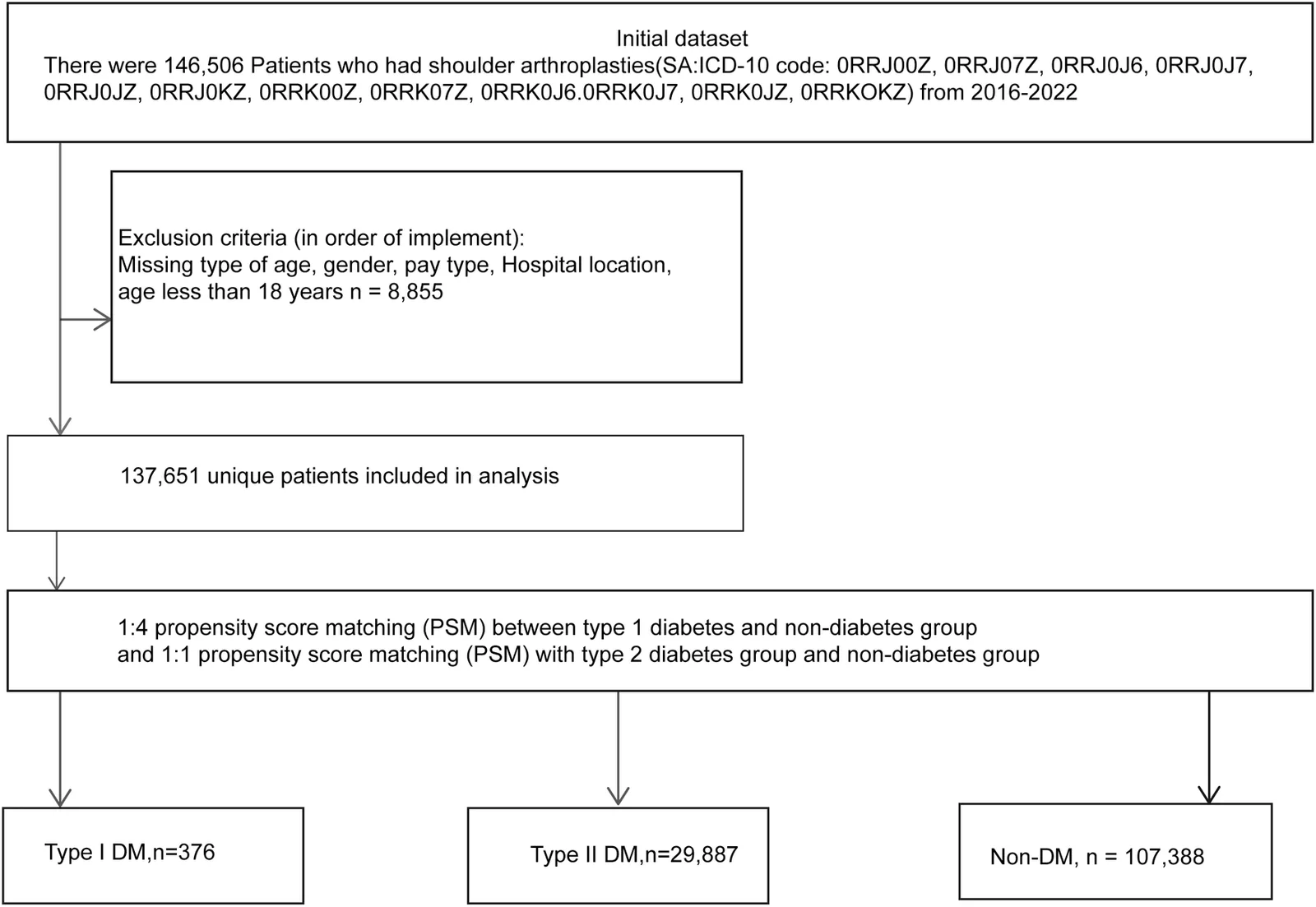
Flow diagram of the study selection process. ICD-10, International Classification of Diseases, Clinical Modification.

After excluding incomplete records and patients aged younger than 18 years, a final analytic cohort of 137,651 cases was established (Figure 1). The NIS supports coding for diabetes mellitus (DM) in the absence of diabetic complications. Accordingly, patients with diabetes-related complications and those with both type 1 and type 2 DM were excluded to enhance homogeneity among patients with diabetes undergoing shoulder arthroplasty.

Eligible patients were categorized into three groups: patients with T1DM (E109), patients with T2DM (E119), and patients without diabetes. Patient-level characteristics included sex, age group, race, insurance type, and comorbidity burden. Hospital-level characteristics included bed size, teaching status, and geographic location.

The primary endpoint was the incidence of multiple in-hospital complications following shoulder arthroplasty, including surgical site or periprosthetic joint infection, acute blood loss, acute kidney injury, pneumonia or acute respiratory failure, urinary tract infection, prolonged invasive mechanical ventilation, and blood transfusion. Additionally, 34 preoperative comorbidities with potential impact on surgical outcomes were evaluated (Table 1) and integrated into the statistical database.

**Table 1:** Demographics, hospital, and operative characteristics as well as comorbidities analyzed.

Variables	Specific variables
Demographics	Age group (18–59, 60–69, 70–79, >80), gender (male and female), race (White, Black, Hispanic, Asian or Pacific Islander, Native American and Other), income (lowest quartile, quartile 2, quartile 3, highest quartile), type of insurance (Medicare, Medicaid, Private insurance, Self-pay, Other).
Hospital characteristics	Admission status (elective or non-elective), transfer-in status (no transfer-in or any transfer-in from another healthcare facility), bed size of hospital (large, medium, small), teaching status of hospital (teaching, non-teaching), location of hospital (urban, rural), region of hospital (Northeast, Midwest or North Central, South, West).
Operative variables	Anatomic total shoulder replacement, Reverse total shoulder replacement, Partial shoulder replacement,Reverse Arthroplasty
Comorbidities	Osteoarthritis, rotator cuff tear, proximal humerus fracture, avascular necrosis, anticoagulant, steroids, osteoporosis, drug abuse, tobacco use, obesity, dyslipidemia, coagulopathyr, hypertension, congestive hearts failure, valvular disease,peripheral vascular disease, pulmonary circulation disorders, cerebrovascular disease,dementia, epilepsy,paralysis,other nervous system,mental disorder,sleep apnea syndrome,chronic obstructive pulmonary disease,chronic kidney disease,liver disease,peptic ulcer,malignancy.

Admission status was defined using the HCUP-NIS ELECTIVE data element and categorized as elective (ELECTIVE = 1) or non-elective (ELECTIVE = 0). Transfer-in status was assessed separately using the HCUP-NIS TRAN_IN data element and was not considered a category of admission status. Transfer-in status was dichotomized as no transfer-in (TRAN_IN = 0) vs. any transfer-in from another healthcare facility (TRAN_IN = 1 or 2). The latter category included transfers from either a different acute-care hospital or another type of healthcare facility.

### Data analysis

Statistical analyses were performed using R version 3.5.3 (R Foundation for Statistical Computing, Vienna, Austria) and IBM SPSS Statistics version 25.0 (IBM Corp., Armonk, NY, USA). A two-sided *α* = 0.05 was defined as the threshold for statistical significance (*P* < 0.05).

Continuous variables were summarized as medians and interquartile ranges, and categorical variables as frequencies and percentages. Baseline characteristics were compared across the three unmatched groups using the Kruskal–Wallis test for continuous variables and Pearson's chi-square test for categorical variables. The *P* values reported in Tables 2, 3 represent overall comparisons across the three groups. Prespecified pairwise comparisons between T1DM and no diabetes, T2DM and no diabetes, and T1DM and T2DM were performed in the subsequent regression and propensity score-matched outcome analyses.

**Table 2:** Demographic, hospital, and surgical characteristics of patients undergoing shoulder replacement surgery before propensity score matching.

Characteristics	Number(%)			Overall *P* value
	Non-DM	Type I DM	Type II DM	
Total	107,388	376	29,887	
Age	70.0 (64.0, 76.0)	67.0 (60.0, 72.0）	71.0 (65.0, 76.0)	<0.001
Age group				<0.001
18–59	15,059 (14.0)	88 (23.4)	3,226 (10.8)	
60–69	35,280 (32.9)	147 (39.1)	10,209 (34.2)	
70–79	41,633 (38.8)	116 (30.9)	12,635 (42.3)	
>80	15,416 (14.4)	25 (6.6)	3,817 (12.8)	
Female	61,342 (57.1)	233 (62.0)	16,655 (55.7)	<0.001
Race				<0.001
White	96,451 (89.8)	332 (88.3)	24,937 (83.4)	
Black	4,304 (4.0)	18 (4.8)	2,100 (7.0)	
Hispanic	3,981 (3.7)	19 (5.1)	1,888 (6.3)	
Asian/PI	583 (0.5)	1 (0.3)	236 (0.8)	
Native	355 (0.3)	1 (0.3)	169 (0.6)	
Other	1,714 (1.6)	5 (1.3)	557 (1.9)	
Median household income				<0.001
Q1	22,937 (21.4)	94 (25.0)	7,864 (26.3)	
Q2	29,201 (27.2)	99 (26.3)	8,840 (29.6)	
Q3	29,516 (27.5)	95 (25.3)	7,740 (25.9)	
Q4	25,734 (24.0)	88 (23.4)	5,443 (18.2)	
Shoulder Replacement Surgery Type				<0.001
Anatomic total shoulder replacement	33,760 (31.4)	94 (25.0)	7,419 (24.8)	
Reverse total shoulder replacement	66,812 (62.2)	237 (63.0)	20,545 (68.7)	
Partial shoulder replacement	6,148 (5.7)	41 (10.9)	1,688 (5.6)	
Reverse Arthroplasty	668 (0.6)	4 (1.1)	235 (0.8)	
Primary payer				<0.001
Medicare	75,280 (70.1)	249 (66.2)	22,040 (73.7)	
Medicaid	3,736 (3.5)	23 (6.1)	1,041 (3.5)	
Private	23,053 (21.5)	89 (23.7)	5,265 (17.6)	
Self-pay/Other	5,319 (5.0)	15 (4.0)	1,541 (5.2)	
Admission status				<0.001
Elective	99,421 (92.6)	325 (86.4)	27,046 (90.5)	
Non-elective	7,967 (7.4)	51 (13.6)	51 (13.6)	
Transfer-in status				<0.001
No transfer-in	106,758 (99.4)	369 (98.1)	29,643 (99.2)	
Any transfer-in from another healthcare facility	630 (0.6)	7 (1.9)	244 (0.8)	
Hospital bed size				0.013
Small	34,070 (31.7)	111 (29.5)	9,188 (30.7)	
Medium	28,583 (26.6)	105 (27.9)	7,974 (26.7)	
Large	44,735 (41.7)	160 (42.6)	12,725 (42.6)	
Region of hospital				<0.001
Northeast	17,678 (16.5)	56 (14.9)	4,306 (14.4)	
Midwest	27,495 (25.6)	95 (25.3)	8,365 (28.0)	
South	40,299 (37.5)	145 (38.6)	12,249 (41.0)	
West	21,916 (20.4)	80 (21.3)	4,967 (16.6)	
Hospital teaching status				0.001
Rural	8,731 (8.1)	34 (9.0)	2,625 (8.8)	
Urban Non-teaching	26,692 (24.9)	75 (19.9)	7,477 (25.0)	
Urban Teaching	71,965 (67.0)	267 (71.0)	19,785 (66.2)	

Admission status was defined using the HCUP-NIS ELECTIVE data element (1 = elective; 0 = non-elective). Transfer-in status was derived separately from the HCUP-NIS TRAN_IN data element and dichotomized as no transfer-in (TRAN_IN = 0) vs. any transfer-in from another healthcare facility (TRAN_IN = 1 or 2). TRAN_IN = 1 indicates transfer from a different acute-care hospital, whereas TRAN_IN = 2 indicates transfer from another type of healthcare facility. Transfer-in status was not considered a category of admission status and did not represent discharge disposition. Percentages were calculated within each diabetes group. A single overall *P* value is reported for each categorical variable. Non-DM, non- diabetes- mellitus; type I DM indicates type I diabetes mellitus; type II DM, type II diabetes mellitus.

**Table 3:** Comorbidities in patients undergoing shoulder replacement surgery before propensity score matching.

Comorbidities	Number (%)			Overall *P* value
	Non-DM	Type I DM	Type II DM	
Osteoarthritis	79,309 (73.9)	214 (56.9)	20,807 (69.6)	<0.001
Rotator cuff tear	31,271 (29.1)	72 (19.1)	9,056 (30.3)	<0.001
Proximal humerus fracture	11,092 (10.3)	112 (29.8)	4,274 (14.3)	<0.001
Avascular necrosis	2,301 (2.1)	13 (3.5)	508 (1.7)	<0.001
Anticoagulant	8,174 (7.6)	45 (12.0)	3,430 (11.5)	<0.001
Steroids	3,421 (3.2)	10 (2.7)	1,168 (3.9)	<0.001
Osteoporosis	7,022 (6.5)	32 (8.5)	1,527 (5.1)	<0.001
Drug abuse	1,108 (1.0)	4 (1.1)	286 (1.0)	0.519
Tobacco use	9,472 (8.8)	42 (11.2)	2,512 (8.4)	0.020
Obesity	21,044 (19.6)	97 (25.8)	10,323 (34.5)	<0.001
Dyslipidemia	46,630 (43.4)	231 (61.4)	19,511 (65.3)	<0.001
Electrolyte disorder	6,339 (5.9)	49 (13.0)	2,464 (8.2)	<0.001
Thyroid disorders	19,553 (18.2)	125 (33.2)	6,392 (21.4)	<0.001
Weight loss	568 (0.5)	2 (0.5)	164 (0.5)	0.917
Autoimmune	12,101 (11.3)	49 (13.0)	3,364 (11.3)	0.556
Anemia	5,543 (5.2)	35 (9.3)	2,000 (6.7)	<0.001
Coagulopathy	2,227 (2.1)	10 (2.7)	832 (2.8)	<0.001
Hypertension	58,749 (54.7)	217 (57.7)	19,535 (65.4)	<0.001
Congestive hearts failure	4,174 (3.9)	35 (9.3)	2,772 (9.3)	<0.001
Valvular disease	4,240 (3.9)	10 (2.7)	1,362 (4.6)	<0.001
Peripheral Vascular Disease	3,559 (3.3)	15 (4.0)	836 (2.8)	<0.001
Pulmonary circulation disorders	1,106 (1.0)	4 (1.1)	473 (1.6)	<0.001
Cerebrovascular disease	1,321 (1.2)	4 (1.1)	572 (1.9)	<0.001
Dementia	1,864 (1.7)	11 (2.9)	638 (2.1)	<0.001
Epilepsy	2,004 (1.9)	11 (2.9)	515 (1.7)	0.077
Paralysis	1,006 (0.9)	5 (1.3)	404 (1.4)	<0.001
Other nervous system	5,941 (5.5)	30 (8.0)	2,024 (6.8)	<0.001
Mental disorder	36,570 (34.1)	144 (38.3)	11,036 (36.9)	<0.001
Sleep apnea syndrome	16,606 (15.5)	83 (22.1)	8,390 (28.1)	<0.001
Chronic obstructive pulmonary disease	9,909 (9.2)	31 (8.2)	3,996 (13.4)	<0.001
Chronic kidney disease	7,507 (7.0)	81 (21.5)	5,093 (17.0)	<0.001
Liver disease	2,242 (2.1)	13 (3.5)	1,095 (3.7)	<0.001
Peptic ulcer	339 (0.3)	2 (0.5)	95 (0.3)	0.757
Malignancy	1,735 (1.6)	7 (1.9)	549 (1.8)	0.029

Non-DM, non-diabetes-mellitus; type I DM, type I diabetes mellitus; type II DM, type II diabetes mellitus.

Propensity score matching (PSM) was used to reduce measured confounding. Because the T1DM cohort was small (*n* = 376), 1:4 matching was used for comparisons involving T1DM to improve precision by drawing on the larger comparator pools while retaining adequate covariate balance. Because the T2DM cohort was substantially larger (*n* = 29,887), 1:1 matching with the group without diabetes was sufficient to achieve balance and common support. Accordingly, the matching ratios were 1:4 for T1DM vs. no diabetes and T1DM vs. T2DM, and 1:1 for T2DM vs. no diabetes. Covariate balance was assessed using absolute standardized mean differences, with a value < 0.10 indicating adequate balance.

**Figure 2:**
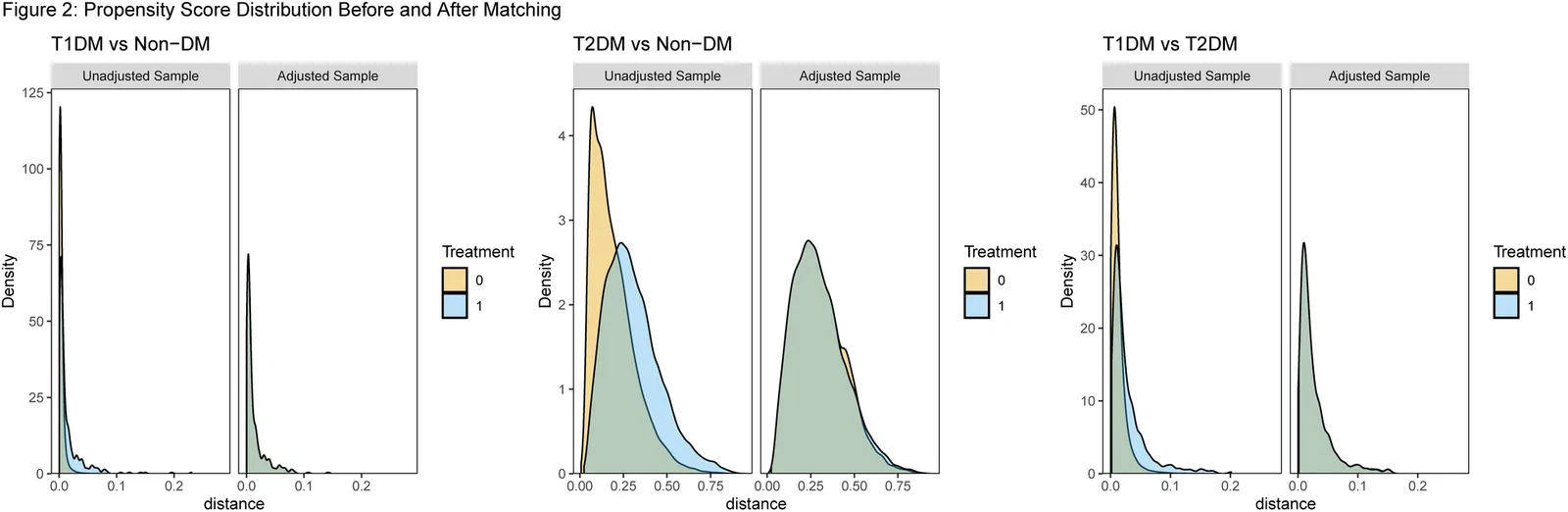
Propensity score distribution before and after propensity score matching (PSM).

The PSM model incorporated all available covariates from the NIS: age group, sex, race, median household income, insurance type, binary admission status (elective vs. non-elective), binary transfer-in status (no transfer-in vs. any transfer-in), hospital bed size, teaching status, location, and census region, to reduce baseline confounding on study endpoints. Balance after PSM is displayed in Figure 2. Outcome analyses were subsequently performed within each propensity score-matched cohort.

Binary postoperative outcomes were analyzed using logistic regression models and are reported as odds ratios (ORs) with 95% confidence intervals (CIs). Length of stay and total hospital charges were log-transformed and analyzed using linear regression, with exponentiated coefficients reported as geometric mean ratios (GMRs). Pairwise analyses compared T1DM vs. non-DM, T2DM vs. non-DM, and T1DM vs. T2DM. Following PSM, Firth logistic regression was used for binary outcomes, whereas log-linear regression was used for length of stay and total hospital charges.

## Results

From 2016 to 2022, a total of 137,651 patients underwent shoulder arthroplasty, including 107,388 (77.99%) patients without diabetes, 376 (0.3%) patients with type 1 diabetes mellitus (T1DM), and 29,887 (21.71%) patients with type 2 diabetes mellitus (T2DM). Before PSM, overall comparisons across the three unmatched groups showed statistically significant differences in most demographic, hospital, surgical, and comorbidity characteristics (Tables 2, 3). Group-specific distributions are presented descriptively in the tables. After PSM, 376 patients with T1DM were matched with 1,504 patients without diabetes, 29,887 patients with T2DM were matched with 29,887 patients without diabetes, and 376 patients with T1DM were matched with 1,504 patients with T2DM.

### Comparison between patients without diabetes and patients with T1DM

Before PSM, significant differences were observed in demographic, hospital, and surgical characteristics between T1DM and patients without diabetes (all *P* < 0.05). Patients with T1DM were younger, with a higher proportion in the 60–69 age group and a lower proportion in the over-80 age group (*P* < 0.001). The T1DM group had a higher percentage of female patients (*P* < 0.001) and a higher prevalence of Black and Hispanic patients (*P* < 0.001). There were significant differences in median household income between the two groups (*P* < 0.001). Differences were also found in the distribution of surgical types, with a higher rate of partial shoulder arthroplasty in the T1DM group (*P* < 0.001). Significant differences were detected in primary payer, admission type, hospital bed size, hospital region, and teaching hospital status between the two groups (*P* < 0.05) (Table 2).

Regarding comorbidities, Patients with T1DM had higher prevalence rates of osteoarthritis, rotator cuff tear, proximal humeral fracture, anticoagulant use, osteoporosis, smoking, obesity, dyslipidemia, electrolyte imbalance, thyroid disease, anemia, coagulopathy, hypertension, congestive heart failure, pulmonary circulatory disorders, cerebrovascular disease, dementia, paralysis, other neurological diseases, mental disorders, sleep apnea syndrome, chronic kidney disease, liver disease, and malignant tumors (all *P* < 0.05). No significant differences were found in substance abuse, weight loss, autoimmune diseases, epilepsy, or peptic ulcer (*P* > 0.05). After PSM, all baseline variables were well balanced between groups (Table 3).

**Figure 3:**
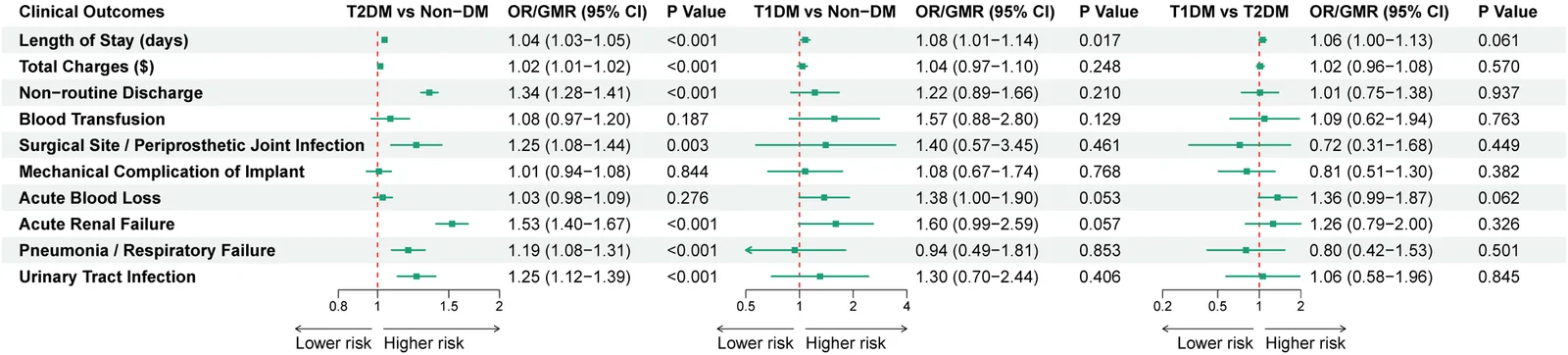
Forest plot showing the risk of postoperative clinical outcomes in patients with T1DM, T2DM, and non-diabetic controls after propensity score matching (PSM).

In univariate and multivariate analyses, most medical and surgical complications did not differ significantly between groups. Univariate analysis showed a higher incidence of prolonged length of hospital stay in Patients with T1DM (*P* = 0.017) (Figure 3). In multivariate analysis, no significant differences were observed in non-routine discharge and increased hospital costs between non-DM and Patients with T1DM.

### Comparison between patients with T1DM and T2DM

In the direct 1:4 propensity score-matched comparison between patients with T1DM and T2DM, no statistically significant differences were observed in the evaluated postoperative outcomes (all *P* > 0.05) (Figure 3).

### Comparison between patients without diabetes and patients with T2DM

Before PSM, Patients with T2DM were significantly older, with a higher proportion in the 70–79 age group and a lower proportion in the 18–59 age group (*P* < 0.001). The T2DM group had a higher percentage of male patients (*P* < 0.001) and a higher prevalence of Black and Hispanic patients (*P* < 0.001). Patients with T2DM had a lower median household income (*P* < 0.001). Patients with T2DM were more likely to undergo reverse total shoulder arthroplasty and less likely to undergo anatomic total shoulder arthroplasty (*P* < 0.001). The T2DM group had a higher rate of Medicare as the primary payer and a lower rate of private insurance (*P* < 0.001), as well as a lower rate of elective admission (*P* < 0.001). Significant differences were found in hospital bed size, region, and teaching status (*P* < 0.05) (Table 2).

Almost all comorbidities were more prevalent in Patients with T2DM (*P* < 0.001), including osteoarthritis, rotator cuff tear, proximal humeral fracture, anticoagulant use, steroid use, obesity, dyslipidemia, electrolyte imbalance, thyroid disease, anemia, coagulopathy, hypertension, congestive heart failure, valvular disease, pulmonary circulatory disorders, cerebrovascular disease, dementia, paralysis, other neurological diseases, mental disorders, sleep apnea syndrome, chronic obstructive pulmonary disease, chronic kidney disease, and liver disease. No significant differences were found in weight loss or substance abuse (*P* > 0.05). After matching, most baseline characteristics were balanced, and all baseline variables were well balanced between groups (Table 3).

The incidence rates of urinary tract infection, acute renal failure, surgical site/periprosthetic joint infection, and pneumonia/acute respiratory failure were significantly higher in Patients with T2DM (*P* < 0.05). After PSM, Patients with T2DM had significantly higher risks of pneumonia/acute respiratory failure (*P* < 0.001), surgical site/periprosthetic joint infection (*P* = 0.003), urinary tract infection (*P* < 0.001), and acute renal failure (*P* < 0.001). Both univariate and multivariate analyses revealed significantly longer length of hospital stay, higher rates of non-routine discharge, and increased hospital costs in Patients with T2DM (Figure 3).

## Discussion

This study provides new national evidence regarding the perioperative burden of shoulder arthroplasty in patients with different types of diabetes. With the continued rise in diabetes prevalence, an increasing number of patients with complex metabolic diseases are expected to undergo shoulder arthroplasty, making the identification of diabetes-specific perioperative risks of significant clinical importance. In this NIS-based analysis, we observed significant differences in demographic characteristics, hospital burden, comorbidities, and perioperative outcomes among patients with type 1 diabetes, type 2 diabetes, and those without diabetic complications. These findings suggest that diabetes is not a single risk state, and that subtypes and comorbidities of diabetes may influence short-term surgical outcomes after shoulder arthroplasty. This study underscores the need for more individualized preoperative risk stratification and perioperative management for this growing patient population. The direct matched comparison between T1DM and T2DM showed no statistically significant differences, although the relatively small T1DM cohort limited the precision of these estimates.

Recent evidence from lower-extremity arthroplasty supports our findings. A 2024 NIS study reported that diabetes was associated with prolonged hospitalization, higher hospital charges, non-routine discharge, and infection after total hip arthroplasty (15), while a 2024 meta-analysis found increased risks of periprosthetic joint infection, pneumonia, and urinary tract infection after total knee arthroplasty among patients with diabetes (16).

Pneumonia and acute respiratory failure were among the significant perioperative outcomes associated with T2DM in our study cohort. These pulmonary events have significant clinical implications. Specifically, patients with chronic obstructive pulmonary disease (COPD) and metabolic syndrome such as diabetes mellitus had a higher risk of postoperative pneumonia during shoulder arthroplasty, suggesting that decreased cardiopulmonary reserve and adverse metabolic status may make patients more susceptible to postoperative pulmonary decompensation (17–19). From a pathophysiological perspective, diabetes and perioperative hyperglycemia impair immune and inflammatory responses, thereby increasing the risk of postoperative infection and subsequent respiratory deterioration (20, 21). Results showed that surgical site/periprosthetic infection was one of the significant perioperative complications in our cohort. Periprosthetic shoulder infection is a devastating complication with significant diagnostic challenges, and the shoulder is often colonized by low-virulence pathogens such as Propionibacterium acnes, which can delay detection (6, 17, 22). In patients with diabetes, our observations are consistent with previous shoulder arthroplasty literature, which suggests that diabetes is associated with an increased risk of infection, and that perioperative glycemic load is associated with a progressive increase in deep postoperative infections (6, 23). Although an earlier registry-based study indicated that isolated diabetes was not independently associated with deep infection, recent evidence suggests that glycemic control and overall metabolic risk profiles may be more informative than a simple yes/no diabetes classification (24–26). Therefore, careful preoperative risk assessment, optimization of relevant comorbidities, close wound monitoring, early assessment of occult infection, preoperative pulmonary assessment, and early postoperative respiratory support are particularly important for patients with diabetes undergoing shoulder arthroplasty.

This study found that urinary tract infection (UTI) and acute renal failure were significant perioperative complications in our cohort. A clinical study found UTI to be a common complication after joint replacement surgery, and diabetes in shoulder replacement patients was associated with an increased risk of postoperative UTI and renal insufficiency (27, 28). Furthermore, previous shoulder replacement studies have shown that patients with a higher comorbidity burden, such as women, those with metabolic syndrome, or those with chronic kidney disease, are more prone to postoperative UTIs and renal complications (29–31). From a pathophysiological perspective, diabetes-related immune dysfunction, perioperative hyperglycemia, catheter exposure, and limited mobility may all contribute to postoperative UTI, while pre-existing diabetic nephropathy, perioperative hemodynamic stress, and susceptibility to nephrotoxic injury may increase the risk of acute kidney injury or acute renal failure (32–36). Therefore, the occurrence of UTI and acute renal failure in this study suggests that patients with diabetes undergoing shoulder arthroplasty require careful perioperative risk management, early catheter removal, adequate hydration, avoidance of nephrotoxic exposures, and close postoperative renal monitoring.

### Limitations

This study has several strengths, including its large sample and evaluation of multiple in-hospital outcomes across diabetes subtypes. Several limitations should also be noted. First, the use of administrative databases such as the NIS inherently carries inherent risks, including inaccurate coding, inter-institutional variability, and missing data for certain clinical variables. Second, critical clinical information is lacking, such as the degree of glycemic control in patients with diabetes, diabetes-related laboratory indicators (e.g., glycated hemoglobin levels), and the exact timing of complications. Third, approximately 6% of patients were excluded due to missing key demographic or outcome variables. Given this relatively small proportion, we consider its potential impact on the study results to be minimal and negligible, although the possibility of introducing selection bias cannot be completely excluded. Fourth, the study design is unable to assess long-term outcomes or the specific mechanisms underlying the observed associations. Fifth, the NIS does not capture patient-reported outcomes or objective postoperative functional measures, including pain, range of motion, strength, or validated shoulder scores. Therefore, we could not determine whether diabetes type was associated with postoperative functional recovery. Prospective longitudinal studies incorporating measures of glycemic control and validated functional outcomes are warranted. In addition, the small T1DM cohort resulted in wide confidence intervals; nonsignificant associations should not be interpreted as evidence of equivalence. Furthermore, while this study initially reveals the associations between type 1/type 2 diabetes and specific complications following shoulder arthroplasty, additional research is needed to clarify the involved pathophysiological processes. Consequently, pathological conditions and related complications that occur only during a long-term observation period are outside the scope of studies using the NIS dataset. Despite the inherent limitations of large administrative databases, these data repositories provide unique opportunities to investigate epidemiological patterns and population-level trends in relatively rare diagnostic categories.

## Conclusion

The results showed that compared with patients without diabetes, type 2 diabetes increased the risk of multiple postoperative complications after shoulder arthroplasty, including surgical site/periprosthetic infection, acute kidney injury, pneumonia/acute respiratory failure, and urinary tract infection. These associations may reflect differences in diabetes subtype and associated comorbidity burden. The findings can provide evidence for precise preoperative risk assessment and individualized perioperative management of patients with diabetes undergoing shoulder arthroplasty.

## Data Availability

The raw data supporting the conclusions of this article will be made available by the authors, without undue reservation.
