# Supplementary material for: Adverse in-hospital outcomes after shoulder arthroplasty by diabetes status: a retrospective study using the national inpatient sample
Source: Front Med (Lausanne). 2026 Sep 9;13:1885571. doi: 10.3389/fmed.2026.1885571 (PMC13597341; doi:10.3389/fmed.2026.1885571)
Supplement: Supplementary file 1 [file Table1.doc]

**Table 1** Demographics, hospital, and operative characteristics as well as comorbidities analyzed

| **Variables** | **Specific variables** |
| --- | --- |
| **Demographics** | Age group (18-59, 60-69, 70-79, >80), gender (male and female), race (White, Black, Hispanic, Asian or Pacific Islander, Native American and Other), income (lowest quartile, quartile 2, quartile 3, highest quartile), type of insurance (Medicare, Medicaid, Private insurance, Self-pay, Other). |
| **Hospital characteristics** | Admission status (elective or non-elective), transfer-in status (no transfer-in or any transfer-in from another healthcare facility), bed size of hospital (large, medium, small), teaching status of hospital (teaching, non-teaching), location of hospital (urban, rural), region of hospital (Northeast, Midwest or North Central, South, West). |
| **Operative variables** | Anatomic total shoulder replacement, Reverse total shoulder replacement, Partial shoulder replacement,Reverse Arthroplasty |
| **Comorbidities** | Osteoarthritis, rotator cuff tear, proximal humerus fracture, avascular necrosis, anticoagulant, steroids, osteoporosis, drug abuse, tobacco use, obesity, dyslipidemia, coagulopathyr, hypertension, congestive hearts failure, valvular disease,peripheral vascular disease, pulmonary circulation disorders, cerebrovascular disease,dementia, epilepsy,paralysis,other nervous system,mental disorder,sleep apnea syndrome,chronic obstructive pulmonary disease,chronic kidney disease,liver disease,peptic ulcer,malignancy. |
