# Supplementary material for: Adverse in-hospital outcomes after shoulder arthroplasty by diabetes status: a retrospective study using the national inpatient sample
Source: Front Med (Lausanne). 2026 Sep 9;13:1885571. doi: 10.3389/fmed.2026.1885571 (PMC13597341; doi:10.3389/fmed.2026.1885571)
Supplement: Supplementary file 2 [file Table2.doc]

**Table2.**Demographic, hospital, and surgical characteristics of patients undergoing shoulder replacement surgery before propensity score matching.

| **Characteristics** | **Number(%)** | | | **Overall P value** |
| --- | --- | --- | --- | --- |
|  | **Non-DM** | **Type I DM** | **Type II DM** |  |
| Total | 107,388 | 376 | 29,887 |  |
| Age | 70.0 (64.0, 76.0) | 67.0 (60.0, 72.0） | 71.0 (65.0, 76.0) | <0.001 |
| Age Group |  |  |  | <0.001 |
| 18-59 | 15,059 (14.0) | 88 (23.4) | 3,226 (10.8) |  |
| 60-69 | 35,280 (32.9) | 147 (39.1) | 10,209 (34.2) |  |
| 70-79 | 41,633 (38.8) | 116 (30.9) | 12,635 (42.3) |  |
| >80 | 15,416 (14.4) | 25 (6.6) | 3,817 (12.8) |  |
| Female | 61,342 (57.1) | 233 (62.0) | 16,655 (55.7) | <0.001 |
| Race |  |  |  | <0.001 |
| White | 96,451 (89.8) | 332 (88.3) | 24,937 (83.4) |  |
| Black | 4,304 (4.0) | 18 (4.8) | 2,100 (7.0) |  |
| Hispanic | 3,981 (3.7) | 19 (5.1) | 1,888 (6.3) |  |
| Asian/PI | 583 (0.5) | 1 (0.3) | 236 (0.8) |  |
| Native | 355 (0.3) | 1 (0.3) | 169 (0.6) |  |
| Other | 1,714 (1.6) | 5 (1.3) | 557 (1.9) |  |
| Median household income |  |  |  | <0.001 |
| Q1 | 22,937 (21.4) | 94 (25.0) | 7,864 (26.3) |  |
| Q2 | 29,201 (27.2) | 99 (26.3) | 8,840 (29.6) |  |
| Q3 | 29,516 (27.5) | 95 (25.3) | 7,740 (25.9) |  |
| Q4 | 25,734 (24.0) | 88 (23.4) | 5,443 (18.2) |  |
| Shoulder Replacement Surgery Type |  |  |  | <0.001 |
| Anatomic total shoulder replacement | 33,760 (31.4) | 94 (25.0) | 7,419 (24.8) |  |
| Reverse total shoulder replacement | 66,812 (62.2) | 237 (63.0) | 20,545 (68.7) |  |
| Partial shoulder replacement | 6,148 (5.7) | 41 (10.9) | 1,688 (5.6) |  |
| Reverse Arthroplasty | 668 (0.6) | 4 (1.1) | 235 (0.8) |  |

(Continued table)

| **Characteristics** | **Number(%)** | | | **Overall P value** |
| --- | --- | --- | --- | --- |
|  | **Non-DM** | **Type I DM** | **Type II DM** |  |
| Primary payer |  |  |  | <0.001 |
| Medicare | 75,280 (70.1) | 249 (66.2) | 22,040 (73.7) |  |
| Medicaid | 3,736 (3.5) | 23 (6.1) | 1,041 (3.5) |  |
| Private | 23,053 (21.5) | 89 (23.7) | 5,265 (17.6) |  |
| Self-pay/Other | 5,319 (5.0) | 15 (4.0) | 1,541 (5.2) |  |
| Admission status |  |  |  | <0.001 |
| Elective | 99,421 (92.6) | 325 (86.4) | 27,046 (90.5) |  |
| Non-elective | 7,967 (7.4) | 51 (13.6) | 2,841 (9.5) |  |
|  |  |  |  |  |
| Transfer-in status |  |  |  | <0.001 |
| No transfer-in | 106,758 (99.4) | 369 (98.1) | 29,643 (99.2) |  |
| Any transfer-in from another healthcare facility | 630 (0.6) | 7 (1.9) | 244 (0.8) |  |
| Hospital bed size |  |  |  | 0.013 |
| Small | 34,070 (31.7) | 111 (29.5) | 9,188 (30.7) |  |
| Medium | 28,583 (26.6) | 105 (27.9) | 7,974 (26.7) |  |
| Large | 44,735 (41.7) | 160 (42.6) | 12,725 (42.6) |  |
| Region of hospital |  |  |  | <0.001 |
| Northeast | 17,678 (16.5) | 56 (14.9) | 4,306 (14.4) |  |
| Midwest | 27,495 (25.6) | 95 (25.3) | 8,365 (28.0) |  |
| South | 40,299 (37.5) | 145 (38.6) | 12,249 (41.0) |  |
| West | 21,916 (20.4) | 80 (21.3) | 4,967 (16.6) |  |
| Hospital teaching status |  |  |  | 0.001 |
| Rural | 8,731 (8.1) | 34 (9.0) | 2,625 (8.8) |  |
| Urban Non-teaching | 26,692 (24.9) | 75 (19.9) | 7,477 (25.0) |  |
| Urban Teaching | 71,965 (67.0) | 267 (71.0) | 19,785 (66.2) |  |

*Values in boldface indicate statistical significance (P<0.05).

Admission status was defined using the HCUP-NIS ELECTIVE data element (1 = elective; 0 = non-elective). Transfer-in status was derived separately from the HCUP-NIS TRAN_IN data element and dichotomized as no transfer-in (TRAN_IN = 0) versus any transfer-in from another healthcare facility (TRAN_IN = 1 or 2). TRAN_IN = 1 indicates transfer from a different acute-care hospital, whereas TRAN_IN = 2 indicates transfer from another type of healthcare facility. Transfer-in status was not considered a category of admission status and did not represent discharge disposition. Percentages were calculated within each diabetes group. A single overall P value is reported for each categorical variable. Non-DM, non- diabetes- mellitus ; type I DM indicates type I diabetes mellitus; type II DM, type II diabetes mellitus.
