# Supplementary material for: Adverse in-hospital outcomes after shoulder arthroplasty by diabetes status: a retrospective study using the national inpatient sample
Source: Front Med (Lausanne). 2026 Sep 9;13:1885571. doi: 10.3389/fmed.2026.1885571 (PMC13597341; doi:10.3389/fmed.2026.1885571)
Supplement: Supplementary file 3 [file Table3.doc]

**Table3.**Comorbidities in patients undergoing shoulder replacement surgery before propensity score matching.

| **Comorbidities** | **Number (%)** | | |  |
| --- | --- | --- | --- | --- |
|  | **Non-DM** | **Type I DM** | **Type II DM** | **Overall P value** |
| Osteoarthritis | 79309 (73.9) | 214 (56.9) | 20807 (69.6) | <0.001 |
| Rotator cuff tear | 31271 (29.1) | 72 (19.1) | 9056 (30.3) | <0.001 |
| Proximal humerus fracture | 11092 (10.3) | 112 (29.8) | 4274 (14.3) | <0.001 |
| Avascular necrosis | 2301 (2.1) | 13 (3.5) | 508 (1.7) | <0.001 |
| Anticoagulant | 8174 (7.6) | 45 (12.0) | 3430 (11.5) | <0.001 |
| Steroids | 3421 (3.2) | 10 (2.7) | 1168 (3.9) | <0.001 |
| Osteoporosis | 7022 (6.5) | 32 (8.5) | 1527 (5.1) | <0.001 |
| Drug abuse | 1108 (1.0) | 4 (1.1) | 286 (1.0) | 0.519 |
| Tobacco use | 9472 (8.8) | 42 (11.2) | 2512 (8.4) | 0.020 |
| Obesity | 21044 (19.6) | 97 (25.8) | 10323 (34.5) | <0.001 |
| Dyslipidemia | 46630 (43.4) | 231 (61.4) | 19511 (65.3) | <0.001 |
| Electrolyte disorder | 6339 (5.9) | 49 (13.0) | 2464 (8.2) | <0.001 |
| Thyroid disorders | 19553 (18.2) | 125 (33.2) | 6392 (21.4) | <0.001 |
| Weight loss | 568 (0.5) | 2 (0.5) | 164 (0.5) | 0.917 |
| Autoimmune | 12101 (11.3) | 49 (13.0) | 3364 (11.3) | 0.556 |
| Anemia | 5543 (5.2) | 35 (9.3) | 2000 (6.7) | <0.001 |
| Coagulopathy | 2227 (2.1) | 10 (2.7) | 832 (2.8) | <0.001 |
| Hypertension | 58749 (54.7) | 217 (57.7) | 19535 (65.4) | <0.001 |
| Congestive hearts failure | 4174 (3.9) | 35 (9.3) | 2772 (9.3) | <0.001 |
| Valvular disease | 4240 (3.9) | 10 (2.7) | 1362 (4.6) | <0.001 |
| Peripheral Vascular Disease | 3559 (3.3) | 15 (4.0) | 836 (2.8) | <0.001 |
| Pulmonary circulation disorders | 1106 (1.0) | 4 (1.1) | 473 (1.6) | <0.001 |

(Continued table)

| **Comorbidities** | **Number (%)** | | | | |  |
| --- | --- | --- | --- | --- | --- | --- |
|  | **Non-DM** | | **Type I DM** | | **Type II DM** | **Overall P value** |
| Cerebrovascular disease | 1321 (1.2) | 4 (1.1) | | 572 (1.9) | | <0.001 |
| Dementia | 1864 (1.7) | 11 (2.9) | | 638 (2.1) | | <0.001 |
| Epilepsy | 2004 (1.9) | 11 (2.9) | | 515 (1.7) | | 0.077 |
| Paralysis | 1006 (0.9) | 5 (1.3) | | 404 (1.4) | | <0.001 |
| Other nervous system | 5941 (5.5) | 30 (8.0) | | 2024 (6.8) | | <0.001 |
| Mental disorder | 36570 (34.1) | 144 (38.3) | | 11036 (36.9) | | <0.001 |
| Sleep apnea syndrome | 16606 (15.5) | 83 (22.1) | | 8390 (28.1) | | <0.001 |
| Chronic obstructive pulmonary disease | 9909 (9.2) | 31 (8.2) | | 3996 (13.4) | | <0.001 |
| Chronic kidney disease | 7507 (7.0) | 81 (21.5) | | 5093 (17.0) | | <0.001 |
| Liver disease | 2242 (2.1) | 13 (3.5) | | 1095 (3.7) | | <0.001 |
| Peptic ulcer | 339 (0.3) | 2 (0.5) | | 95 (0.3) | | 0.757 |
| Malignancy | 1735 (1.6) | 7 (1.9) | | 549 (1.8) | | 0.029 |

Values in boldface indicate statistical significance (P<0.05).

Non-DM, non-diabetes-mellitus; type I DM, type I diabetes mellitus; type II DM, type II diabetes mellitus.
